# Supplementary material for: User acceptability and perceived impact of a mobile interactive education and support group intervention to improve postnatal health care in northern India: a qualitative study
Source: BMC Med Inform Decis Mak. 2025 Feb 20;25:93. doi: 10.1186/s12911-025-02935-7 (PMC11844055; doi:10.1186/s12911-025-02935-7)
Supplement: Supplementary file 1 — Supplementary Material 1 [file 12911_2025_2935_MOESM1_ESM.docx]

### Supplementary Materials

### Appendix 1. Conceptual Framework of Intervention Context, MeSSSSage Intervention Targets, Outcomes and Anticipated Long-Term Impacts


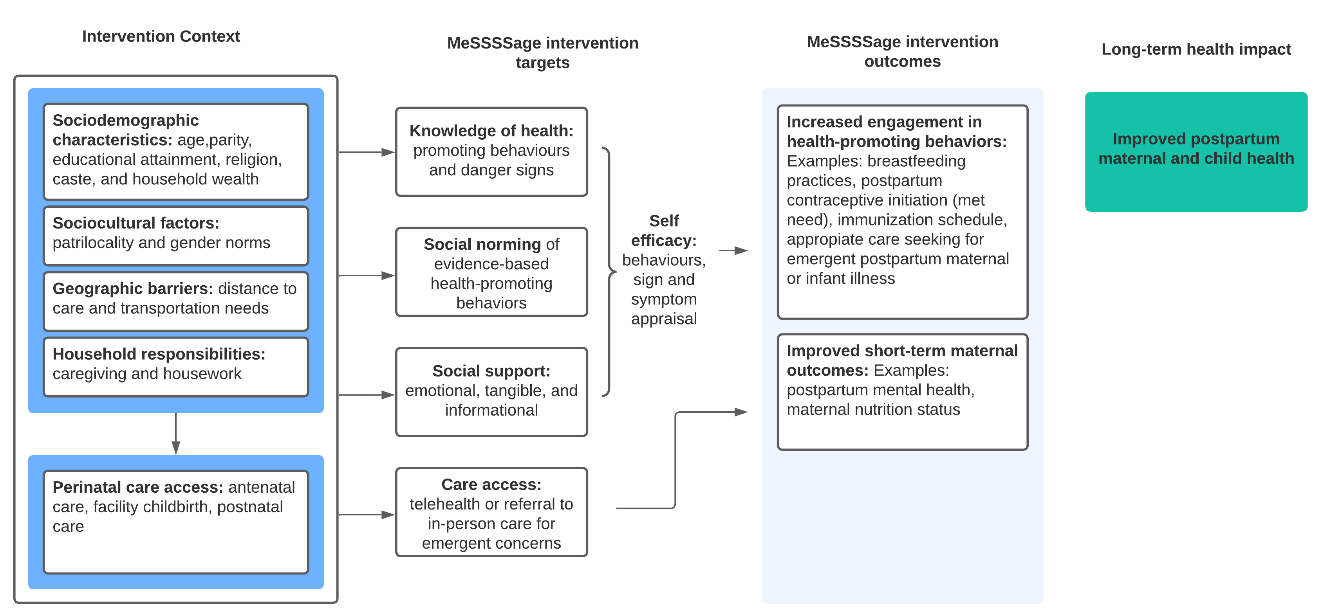


MeSSSSage intervention targets include knowledge and social support. Primary intervention outcomes include increased engagement in health promoting behaviors and improved maternal outcomes. Adapted from El Ayadi et al, 2022 [28].

#### Appendix 2. Educational component of the MeSSSSage intervention.

| Maternal Content | Neonatal Content |
| --- | --- |
| - General Well being - Diet and Nutrition - Iron, folic acid & calcium supplementation - Fetal surveillance - Minor ailments - constipation, low back ache, urgency - COVID appropriate behaviors - Expected date and place of delivery - relevance - Previous antenatal records and investigations - Birth spacing - Delivery preparedness - Psychological issues and fears - Danger signs to watch for - Signs of labor - Maternal hygiene & self-care - Breastfeeding - Sensitization towards responsibility as an expectant/new mother - Kangaroo mother care - Early danger signs after delivery - Perineal care / C- section care - Home hygiene and care after delivery - Self-care - Diet & Nutrition - Mental and emotional health - Family and social support - General postpartum concerns - Postpartum visits - Sexual health - presumption of coitus - Contraceptive methods - Mobile phone for health - Awareness about postnatal physiological changes - Postnatal exercises | - Danger signs in the newborn baby - Breastfeeding - Temperature regulation - Hygiene - Massage - Bathing - Skin rash - Diaper rash - Stooling pattern - Bladder habits - Baby’s sleep - Weight gain and loss pattern - Immunization - Play and communication - Soothing your baby - Abdominal distension - Feeding - Sleep - Developmental milestones - Starting feeds other than breastmilk |

## Appendix 3. In-depth Interview Guide

**MeSSSSage: Second Phase Pilot Study In-depth Interview Guide**

Study ID: __ __ __ __ __ Group Number: __ __ Group Type: A B C D

Date: __ / __ / __ (DD/MM/YY) Start time: __: __ End time: __: __

Interviewer ID: __ __ __

**Introduction:** Thank you so much for participating in this interview. It is important to get your thoughts on your experience in the MeSSSSage intervention because it would help us improve the MeSSSSage intervention for future participants. I have some questions that I will ask you, but please feel free to share any other feedback that you think is relevant or skip any question or end the interview at any time.

1. **Overall – Perceived impact**

First, I would like to ask you a few short questions about the program in general.

|  | Please tell me about your experience so far with the MeSSSSage program.  *Probe: How you were related to the program?*  *Probe: What were your motivations to engage with the program? What were possible barriers to engage with the program?*  *Probe: What do you like about the program? What else?*  *Probe: What do you dislike? What do you think could be improved? What else?*  *Probe: Overall, how satisfied are you with the MeSSSSage program?* |
| --- | --- |
|  | Overall, how would you describe the effect of MeSSSSage program on your experience after giving birth?  *Probe: What information or resources do you take away from participating in it?*  *Probe: Is there anything you are doing differently because participating?*  *Probe: How your experience of motherhood has been affected by participating in MeSSSSage?*  *Probe: How has your perception of breastfeeding changed since participating in MeSSSSage?*  *Probe: Hos has your perception of infant danger signs and seeking care changed since participating in MeSSSSage?*  *Probe: How has your perception of family planning changed since participating in MeSSSSage?* |

1. **Educational component: Use of IVR system or App**

Now I would like to know your perception about the educational aspect of the intervention.

*Note for the interviewer: Next questions can be used either for the IVR system or App, except nº4 (only App).*

|  | First, how do you receive the education content each week? |
| --- | --- |
|  | ***[This question only for those who used the mobile App]***  Please tell me about your overall experience receiving the health education content.  *Probe: How do you feel using the app?*  *Probe: How easy was to learn how to use it? Were you able to use the app immediately?*  *Probe: What do you like about it?*  *Probe: What do you think could be improved?*  *Probe: Did you have any problem while downloading the app?*  *Probe: Did you have any problem using the app after downloading it? (Examples: slow at times, icons or menu unclear, small size of buttons/icons/content)*  *Probe: How did you find the app looks? Did you find the app visually appealing?*  *Probe: Is there anything else you would like to add or change?* |
|  | How do you feel about the health education content shared?  *Probe: How well did you understand the information presented? Was the level of detail too much, too little or the right amount? What could have helped you understand it better?*  *Probe: Were there other topics you would have liked to have had covered? What maternal topics? What neonatal topics?*  *Probe: How relevant was the material to you?*  *Probe: Is there anything you are doing differently after hearing the educational material?*  *Probe: Overall, how useful has been the educational content provided?* |

*Note for the interviewer: The next two sections (page 3 and 4) are about the group calls and the WhatsApp group chat. If participant is only using IVR or the mobile app (Group C and D), move to Closure (page 5).*

1. **Participation and dynamics. Group calls *[For those who participate through zoom]***

We would like to know your perceptions and thoughts about the group calls.

|  | Please tell me about your overall experience with the group calls.  *Probe: What do you like? What else?*  *Probe: What you dislike What else?*  *Probe: What do you think could be improved?*  *Probe: How helpful have videos and photographs explained some topics during the group calls?*  *Probe: In your opinion, what advantages were there to having other participants on the calls? What disadvantages were there?*  *Probe: How relevant has been participating in the group calls?*  *Probe: Is there anything you are doing differently after participating in the group calls?* |
| --- | --- |
|  | How comfortable do you feel participating in the group calls?  *Probe: Did you ever have a question or want to say something but didn’t feel comfortable to ask?*  *Probe: What would have made you feel more comfortable participating in the group calls?*  *Probe: In the future, what can we do differently to help make mothers more comfortable to ask questions?* |
|  | What could have increased the level of connection that you felt with the other group call participants?  *Probe: How do you feel about the interaction with other participants?*  *Probe: Have you been able to make friendships? How?*  *Probe: How can call moderators help to establish better relationships between the participants?* |
|  | What challenges did you experience to participating in the group calls?  *Probe: Comfort level, interest level, time/other responsibilities, phone access, support of household members, network*  *Probe: How could [barriers mentioned] have been overcome to increase engagement?* |

1. **Participation and dynamics. WhatsApp groups**

We would like to know your perceptions and thoughts about the WhatsApp group chat.

|  | Please tell me about your overall experience with the WhatsApp group chat.  *Probe: What do you like? What else?*  *Probe: What you dislike? What do you think could be improved? What else?*  *Probe: How helpful have videos and photographs explained some topics on the WhatsApp group chat?*  *Probe: How useful has been participating in the WhatsApp* *group chat?*  *Probe: How connected have you feel with other participants?* |
| --- | --- |
|  | How comfortable did you feel participating in the group WhatsApp conversations?  *Probe: Did you ever have a question or want to say something in the group WhatsApp but didn’t feel comfortable? Why?*  *Probe: What would have made you feel more comfortable participating in the group text conversations?*  *Probe: In the future, what can we do differently to help make mothers more comfortable to use the WhatsApp group?* |
|  | What could have increased the level of connection that you felt with the other participants in the WhatsApp chat group? |
|  | What challenges did you experience to participating in the WhatsApp chat group?  *Probe: Comfort level, interest level, time/other responsibilities, phone access, support of household members, network, privacy issues, literacy*  *Probe: How could [barriers mentioned] have been overcome to increase engagement?* |

1. **Closure**

Finally, we would want to ask about your family’s opinion on MeSSSSage and any other suggestions from you to improve the program for future mothers.

|  | What do you feel are your family perceptions on the MeSSSSage program?  *Probe: What types of comments have you received from your family members about the program?*  *Probe: What they like? What else?*  *Probe: What they dislike?*  *Probe: Is there anything we could do different to help mother’s families engage with the program?*  *Probe:* *Did you or any of your family members have concerns about your participation in the program? If so, how did you try to overcome this?* |
| --- | --- |
|  | Any final suggestions, recommendations, or thoughts you would like to share with us? |

Thank you so much for your time.

***Construction of in-depth interview questions informed by the realist evaluation model and the COM-B model.***

| ***Aim*** | **Examples of in-depth interview questions** |
| --- | --- |
| Explore user perspectives on barriers and facilitators to engaging with the MeSSSSage intervention. | What challenges did you experience to participating in the group calls?  *Probe: Comfort level, interest level (Actors), time/other responsibilities, phone access, support of household members, network (Context)*    Please tell me about your overall experience receiving the health education content.  *Probe: Did you have any problem using the app after downloading it? Examples: slow at times, icons or menu unclear, small size of buttons/icons/content (Intervention).* |
| Explore perceived impact of the MeSSSSage intervention. | Overall, how would you describe the effect of MeSSSSage program on your experience after giving birth?  *Probe: What information or resources do you take away from participating in it? (Opportunity)*  *Probe: Is there anything you are doing differently because participating? (Capability - Motivation)* |

Examples of *in-depth interview questions* for data collection by aim, informed by the models previously described. Own elaboration, based on the Realist evaluation framework from Kabongo et al, 2021 [30].

## Appendix 4. Themes and quotes obtained from the in-depth interviews

*Acceptability: Satisfaction with the educational and social support component.*

| ***Satisfaction*** | ***Quotes*** |
| --- | --- |
| ***Educational component:***  Usefulness | *“I- Did you face any language barrier? Did you like the content or found it useful during your pregnancy? P- Yes ma’am. It was very useful." (Participant 1, Group call + App + WhatsApp)*  *“Yes ma’am, because of the group, I am able to take care of the baby in a better way.” (Participant 4, Group call + IVR + WhatsApp)*  *"I- Ok, so this was your second child, did you learn anything extra although you had a good experience with your first child? R-Mam, yes, I had the experience, but when we have a new member in the family, it gets tough to take care of all. You taught me how to manage the child and take care of all.” (Participant 7, Group call + IVR + WhatsApp)*  *"I-How has your experience been overall? R- Mam, it is great to speak to all of you and I got to learn many things, and this has been my best experience in motherhood because we don’t have to rush anywhere to know certain things. We save our time and get to learn things at home, so I think it is great talking to the doctors and you all. I shared information with my friend also, the pediatrician calls us weekly, so she knows about her baby and their health.” (Participant 6, Group call + IVR + WhatsApp)*  *“I - What do you think about the content, did you feel that details are too much or too low, do you like the quantity? R- Ok mam, the quantity is neither long nor low, information is accurate, is just to the point, no extra or useless. At some time, we could have felt that it was just a waste, and we could have disconnected the call, but in this group, information is low, but the quality is good, we like to listen. At the start, I seriously used to avoid it, when the phone rang, I used to disconnect it, but one day I picked up over the third call and listened to the call on speaker when I was cooking. That day I liked the information given and then never missed the call again” (Participant 12, Group call + IVR + WhatsApp)*  *“R- Like, mam I have already one baby. At that time, I used to think, “if someone had with me to share my confusion.” I like the things in the group, there is a doctor team, proper guidance and participants are same as us. If we have some problem or confusion, we can contact you. Before we couldn’t go outside, we asked our mother, grandmother, and then did according to their advice. If after this, we didn’t feel relief, then we went to the hospital” (Participant 12, Group call + IVR + WhatsApp)*  *“I – Anything which you didn’t know earlier but after joining the group through app or call, you came to know about it? Or your perception got changed because of it? R- Yes mam, earlier [before the intervention] we couldn’t clear some queries from doctors because they don’t have much time and we also don’t have much time. But in the group or in the app, we are able to clear our doubts, and whenever we feel then we start playing the recording. That is how our doubts get cleared, it was so helpful. I – So you are pretty satisfied. R – Yes mam, I am satisfied, like I get the call from PGI, then I ask my questions and clear them up. I wait with excitement for the 3 pm call” (Participant 14, Group call + App + WhatsApp)* |
| ***Social support component:***  Validation / sharing of motherhood experiences | *“I- Anything you want to talk about the WhatsApp group? Anything you would like us to add? P- Ma’am, not just the child’s but we can also discuss our problems in the group.” (Participant 3, Group call + IVR + WhatsApp)*  *“Mam, you have done enough. Because of you, I get to speak to different mothers, and you always replied to my calls and never missed it, so your efforts are good.” (Participant 8, Group call + IVR + WhatsApp)*  *“So, in the group we do discussions, I hear the IVR messages, they normalize all the things that are ok. Initially, we said one or two months is ok [to rest] then you get normal. Then I thought I was not well for one and half months, what do I do now? Then you said it is ok for six months, take time to heal yourself and body changes, rest and normalize things. Then I feel relaxed as earlier I got panic because of fear, a backache problem, but after this program, I feel comfortable.” (Participant 12, Group call + IVR + WhatsApp)*  *“I – so what do you like most in our group? R- Mam, if someone asks a question, we also get information and knowledge about the same, which medicines should we take and when, and it's helpful” (Participant 14, Group call + App + WhatsApp)* |

*Barriers and facilitators to engage to MeSSSSage*

| ***Barriers*** | ***Quotes*** |
| --- | --- |
| ***From the Context***  Network  Housework  responsibilities  Baby care | *“Sometimes I might not be able to attend them [the calls] on time because my baby has become very naughty these days, she does not let me attend the calls these days” (Participant 1, IVR)*  *"Sometimes I miss out [the call] and sometimes the call gets disconnected and then I don’t get the call back.” (Participant 2, IVR)*  *"It took time [to download the app] as there is an issue of network at our place.*  *I- Ok and what about the videos? R- Some of them were running smoothly and some of them were buffering.” (Participant 11, App)*  *“Mam, the problem was that I am unable to hear your voice and my voice didn’t reach you, even the questions other ladies asked I wasn’t able to hear. I – Have you ever tried to message on what’s app group? R- I had put a question about sugar, but they used to say join the meeting, then you will get the answers” (Participant 15, Group call + App + WhatsApp)*  *“I have missed calls in the past, and I feel awful about that, but being a housewife, we have to take care of many things ourselves, and last time also there was an issue of network range, so these are the reasons.” (Participant 6, Group call + IVR + WhatsApp)*  *"Mam, we live in a village, so I have to look all by myself, the child, the work so I was never active [on the group calls] but when my mother-in-law would take care of my child, I would attend the calls and also there is some network issues." (Participant 10, Group call + App + WhatsApp)*    *“Sometimes in between I had disconnected the call because husband's call comes from behind and if you don't answer then you get scolded” (Participant 12, Group call + App + WhatsApp)*  *“We are two sisters-in-law and we both delivered children with a gap of almost two months, so we have no one to take care of the baby. Our mother-in-law is no more, so one take care of the child and other take care of the household, so we barely get time to see our phones.” (Participant 11, App)*  *“I had attended during pregnancy period only, after delivery I didn’t attend. […] I have two children now elder one is four-year-old and the second one is too little, then his care and household work, I don’t get any time for these meetings.” (Participant 15, Group call + App + WhatsApp)*  *“The reason [why we don’t attend the call] is that we fall asleep, time is ok but sometimes if the baby sleeps late, then we get tired and fall sleep with him” (Participant 13, Group call + IVR + WhatsApp)* |
| ***From the Intervention***  Technological issues | *“Mam we can directly talk to them [the participants] on call sometimes. I do not have any idea about the [WhatsApp] group also as my husband did it, but can we have the contacts of other participants?” (Participant 6, Group call + IVR + WhatsApp)*  *"Actually, ma’am I faced no problem while downloading as I knew we need to go to file first. So, in people’s system it got downloaded in files and some of them [other participants] could not figure that out.” (Participant 1, Group call + App + WhatsApp).*  *"Mam, it took some time [to download the app] and I also got a call from you that I should use another number and then download it.” (Participant 5, App)*  *“Yes mam, earlier I was facing same [problem to download the App] but it was resolved later when I talked to you.” (Participant 14, Group call + App + WhatsApp)*  *“I – So can you tell me where you are having an issue? Is it not open or unable to download? R- Don’t know mam, I have tried many times but still it was not download [the App]” (Participant 16, Group call + App + WhatsApp)*  *“At that time [when using the app] the voice was not clear, sometimes I am unable to hear the voice over the video call [the group call]. I was diabetic at that time, and I put queries but neither get any response nor get proper voice quality, so after that I didn’t join the meeting” (Participant 15, Group call + App + WhatsApp)* |
| ***From the actors***  Participant’s literacy    Level of connection with other participants  Level of comfort:   - Participation of a husband in one group call - Not feeling confident to ask personal questions. | *“I just want to say that you send your voice recording, am not able to read much. I am not able to understand what you had sent [a written message through the group chat]” (Participant 9, Group call + App + WhatsApp)*  *“It has a drawback [the group call]: that involvement of each participant is low, we don’t talk like friends, we are just comparing ourselves with each other, like she has a beautiful baby, so I will not share a picture of my baby. Because of these thoughts, mothers are not involving in the group. If someone is posting a picture, then nobody appreciates her. If someone appreciates and participates in every discussion, then it feels like family. Otherwise, it’s like we are just connected with you only“ (Participant 12, Group call + IVR + WhatsApp)*  *"Once I had [feel uncomfortable]. I wanted to ask something but was unable, later I had to text you […] On that day, her husband attended the call, because she was not present if you remember […] he shared very useful information, I appreciate on father’s involvement. Still, being a female on call or WhatsApp, it breaches the privacy, it was much uncomfortable for me to share personal things in the presence of a male.” (Participant 12, Group call + IVR + WhatsApp)*  *“Mam, I send the query about sex and relationship [directly to the moderator]. I know it is a question of all, l but I didn’t feel comfortable to ask same in front of everyone.” (Participant 13, Group call + IVR + WhatsApp)* |

| ***Facilitators*** | ***Quotes*** |
| --- | --- |
| ***From the context***  Family buy-in   - Partner - Mother-in-law | *"Not just my in-laws but even in my family from my mother’s side everyone said it was a very wise decision that I joined the group” (Participant 1, Group call + App + WhatsApp).*  *"My family is very happy [with the intervention] and when my child had an issue in the eye, they told me to message the doctors of our area in the group or to Dr. X, that they would help us out. My husband is very supportive as well. I- And if we would add your family members to the group, how would they react? R-Mam they will be really happy as they like all your calls and are willing to participate.” (Participant 6, Group call + IVR + WhatsApp)*  *"They [family] were happy as we got all the information at home, and we did not have to run to the hospital. Whenever we messaged, we got the information that instant only as you would reply in the group and pick up calls whenever needed, so it was very much welcomed by my family.” (Participant 8, Group call + IVR + WhatsApp)*    *"He [husband] helped me to download and join the group, and said it is good for you, keep active on the group. I – When you join us on call and chat, then isn’t your family angry with you or do you think it is a waste of time? R- No mam, I usually answer the call in front of my family.” (Participant 9, Group Call + App + WhatsApp)*    *"I – What is the idea or thought of your grand mother-in-law about this call or group? R- She encourage us.” (Participant 9, Group call + App + WhatsApp)*    *" I thought that it might be not that important [to join the calls] but then I joined it and my mother-in-law said that I should do it.” (Participant 10, Group call + App + WhatsApp)*  *“She [mother-in-law] supports me a lot because she knows that instead of taking the baby outside to consult the doctor, it is better to discuss everything over the call and post it in the group. This thing she likes most, that every problem gets instant solution.” (Participant 13, Group call + App + WhatsApp)*  *“They encourage me [the family], because due to Covid we are not able to visit the hospital and the clinic and this makes it better for us, we get all solutions over the call” (Participant 14, Group call + App + WhatsApp)*  *“Even they always said to me [the family], you should join the meeting, you will gain some knowledge, you should contact to them, they are from PGI.” (Participant 15, Group call + App + WhatsApp)* |
| ***From the intervention***    WhatsApp interface (“repeat mode” and use of videos) | *“Mam, you told in the group, make baby movement videos and share them in the group. Then I realized that we need to pay attention to the baby, how active the baby is, according to his movement. I used to play with him. Those videos are nice, and I think it is beneficial for me" (Participant 13, Group call + IVR + WhatsApp)*  *“If we are unable to understand [the videos or audios in WhatsApp], then we play it on repeat mode, and everything is cleared out” (Participant 14, Group call + App + WhatsApp)* |
| ***From the actors***  Intervention moderator’s guidance    Participant’s personal motivation | *"So, she [intervention moderator] explained to me about the Zoom app. How to attend the call. She explained me everything very properly.” (Participant 1, Group call + App + WhatsApp).*  *"I- Have you faced any problem, like how to join the call, how to talk over the call? R - No mam, you had told us how to click, then I click on the link" (Participant 9, Group call + App + WhatsApp)*  *“Mam, I did not have very high expectations for the program, but as soon as it went on, I really learned a lot and it had lots of information. I used to wait for Tuesday for the call.” (Participant 7, Group call + IVR + WhatsApp)*  *“Whenever we get the call, they provide information regarding our health and the baby’s, we like this, and our problem gets solved from your advice. In the app we get all information on how to check baby movement, it motivates us.” (Participant 14, Group call + App + WhatsApp)* |

*Perceived Impact*

| ***Taking care of the baby***    ***Breastfeeding***    ***Family planning***    ***Provide support to community*** | *“I came to know a lot of things [after the calls]. During our first pregnancy, we do not know anything. Through the group I came to know a lot. How to breastfeed, how to take care of the baby, what to use and what not to use for the baby.” (Participant 1, Group call + App + WhatsApp)*  *“Because of the group, I am able to take care of the baby in a better way. […] R- Yes ma’am, like they say we can apply ghee on the naval area of the child but you on the calls mentioned that one should not apply ghee." (Participant 3, Group call + IVR + WhatsApp)*  *“I learned many things. That breast milk is sufficient for the child, and it is a complete food, and the artificial milk should be avoided, and it is very tough to travel when the child is taking artificial milk, so I think breast milk is the best and same was told by the doctors also.” (Participant 8, Group call + IVR + WhatsApp)*  *“R- We all are first in this thing [motherhood] and share all the information in the group, it was good. With your support, I can do the things perfectly as I was unable to do earlier in my first baby. I – Can you give an example of one thing? R- Like– feeding the baby, before I feel pain in breast nipples during feeding, so I consult doctors then they told me brown part of nipples fill full in the baby mouth, then you will not feel pain, and I noticed if he chew on front portion then it is painful for me, and still I followed the same procedure, and many more things I learned" (Participant 12, Group call + IVR + WhatsApp)*  *“R – Mam, my breast milk did not come properly, so I thought to give formula milk. But all said, don’t give formula milk, try to give your breast milk if not came properly, then use a liquid diet. So, I thought about it when doctor said to us, after this, my milk came properly.” (Participant 13, Group call + IVR + WhatsApp)*  *”I - And your family planning perception changed after joining this program?*  *R- you always guide us, that we should take a gap for the next child or use some contraceptive. It is fine we are very much conscious about it and get motivation from your side.” (Participant 12, Group call + IVR + WhatsApp)*    *"I- Ok so did you learn anything from them [group calls, IVR audios], and can you help anyone you know? R-Yes, mam, l learned a lot, and I can give suggestions to people who need help. It was an educational program overall.” (Participant 7, Group call + IVR + WhatsApp)*  *“R - Even my sister-in-law had the same issue, she just fed the baby and put the baby away from herself, she didn’t keep the baby near herself for two months. Then everyone started blaming her, then I told my brother-in-law that these things happen with some mothers, they can’t control their emotions, but you need to take care of her. I – Then his behavior changed? R- Yes, then he used to take my advice and at night she just fed the baby, and then the child slept with him.” (Participant 12, Group call + IVR + WhatsApp)*  *“I- In your neighborhood, friends, family, did they get benefits through you, like you share some information with them? R- Yes mam, in the family, someone as a baby, then I used to tell the mother these things we learned from the program. I –With whom do you share the things? R –Mam, sister-in-law. I- What? R-On baby rashes, she used cream over it, then I told her to use coconut oil.” (Participant 13, Group call + IVR + WhatsApp)*  *I – Ok, ever in your surroundings, in community and neighborhoods someone needed help and you could give help, or were you able to suggest them, in your family and in those near you? R- Mam, regarding which? I – In general, like we provide information to you and you were are able to help others […] R- Yes mam, we share such things, like in our neighborhood there is a young baby, so I share information about her medicines with her mother” (Participant 14, Group call + App + WhatsApp)* |
| --- | --- |
